# Supplementary material for: Modulation of serotonin transporter expression by escitalopram under inflammation
Source: Commun Biol. 2024 Jun 8;7:710. doi: 10.1038/s42003-024-06240-3 (PMC11162477; doi:10.1038/s42003-024-06240-3)
Supplement: Supplementary file 2 — Supplementary Information [file 42003_2024_6240_MOESM2_ESM.pdf]

Supporting Information for

Modulation of Serotonin Transporter Expression by Escitalopram under Inflammation

Sergio Mena[1], Allison Cruikshank[2], Janet Best[3], Nijhout, HF[4], Michael C. Reed[2], Parastoo Hashemi[1*]

[1] Department of Bioengineering, Imperial College London, SW7 2AZ UK

[2] Department of Mathematics, Duke University, Durham, NC, USA

[3] Department of Mathematics, The Ohio State University, Columbus, OH, USA

[4] Department of Biology, Duke University, Durham, NC, USA

Email: phashemi@imperial.ac.uk

This file includes:

- Supplementary Notes 1-5
- Supplementary Figures S1-S5

Full definition of the model

Pharmacokinetics of escitalopram (Supplementary Note 1)

ODE system

$$\frac{dQ_0}{dt} = inj - k_{01}C_0 \quad (1)$$

$$\frac{dQ_1}{dt} = k_{01}Q_0 - (k_{10} + k_{12} + k_{13})Q_1(1 - P_B) + k_{21}Q_2\left(1 - P_B^{brain}\right) + k_{31}Q_3 \quad (2)$$

$$\frac{dQ_2}{dt} = k_{12}Q_1(1 - P_B) - k_{21}Q_2\left(1 - P_B^{brain}\right) \quad (3)$$

$$\frac{dQ_3}{dt} = k_{13}Q_1(1 - P_B) - k_{31}Q_3 \quad (4)$$

Constant parameters

Parameter	Value	Description
k_{01}	0.60*	Rate of transport from peritoneum to plasma (h^{-1})
k_{10}	3	Rate of removal from plasma (h^{-1})
k_{12}	9.90	Rate of transport blood to brain (h^{-1})

k_{21}	2910	Rate of transport brain to blood (h^{-1})
k_{13}	6	Rate of transport blood to periphery (h^{-1})
k_{31}	0.60	Rate of transport periphery to blood (h^{-1})
P_B	0.56	Ratio of escitalopram bound to proteins in plasma
P_B^{brain}	0.15	Ratio of escitalopram bound to proteins in brain

[*]Set to 40% when modelling oral administration of escitalopram.

System variables and initial conditions

Variable	Initial condition	Description
Q_0	0	Escitalopram in peritoneum (µg)
Q_1	0	Escitalopram in plasma (µg)
Q_2	0	Escitalopram in brain (µg)
Q_3	0	Escitalopram in periphery (µg)

Function-defined variables

Escitalopram intraperitoneal injection rate (µg h^{-1})

Function

$$inj(t) = \begin{cases} 0, & t < t_{start} \\ q/t_{inj}, & t_{start} < t < t_{start} + t_{inj} \\ 0, & t > t_{start} + t_{inj} \end{cases}$$

$$q = dose \cdot weight \cdot bioavailability$$

Parameters

q: effective quantity of injected escitalopram into the peritoneum (µg). Dependent on dose given (mg kg^{-1}), weight (mg) and bioavailability.
t_{start}: time at start of injection (h).
t_{inj}: duration of injection (h).
When a train of injections is applied, $t_{start} = t_{start}^0 + n_{inj} \cdot t_{interval}$, where t_{start}^0 is the start time of the first injection (h), n_{inj} is the injection number starting from 0 and $t_{interval}$ is the time between injections (h)

Pharmacokinetics of α-fluoromethylhistidine (Supplementary Note 2)

Pharmacokinetics of α-fluoromethylhistidine were modelled as the four-compartment model modelled in Equations 1-4, and analogous system variables given above, and the following constants:

Constant parameters

Parameter	Value	Description
k_{01}	3.75	FMH rate of transport of peritoneum to plasma (h^{-1})
k_{10}	1.75	FMH rate of removal from plasma (h^{-1})
k_{12}	0.1875	FMH rate of transport blood to brain (h^{-1})
k_{21}	3.5	FMH rate of transport brain to blood (h^{-1})
k_{13}	5	FMH rate of transport blood to periphery (h^{-1})
k_{31}	1.5	FMH rate of transport periphery to blood (h^{-1})
P_B	0.6	Ratio of FMH bound to proteins in plasma
P_B^{brain}	0.15	Ratio of FMH bound to proteins in brain

Serotonin terminal model (Supplementary Note 3)

ODE system

$$\frac{dbtrp}{dt} = TRP_{in} - VTRP_{in}(btrp) - a_1 \cdot (btrp - btrp_0) \tag{5}$$

$$\frac{dbh2}{dt} = inhib_{5HTto5HT}^{syn}\left(G_{5HT}^{*}, G_{5HT,0}^{*}\right) \cdot V_{TPH}(trp, bh4) - V_{DRR}(bh2, NADPH, bh4, NADP) \tag{6}$$

$$\frac{dbh4}{dt} = V_{DRR}(bh2, NADPH, bh4, NADP) - inhib_{5HTto5HT}^{syn}\left(G_{5HT}^{*}, G_{5HT,0}^{*}\right) \cdot V_{TPH}(trp, bh4) \tag{7}$$

$$\frac{dtrp}{dt} = VTRP_{in}(btrp) - inhib_{5HTto5HT}^{syn}\left(G_{5HT}^{*}, G_{5HT,0}^{*}\right) \cdot V_{TPH}(trp, bh4) - V_{pool}\left(trp, trp_{pool}\right) - a_{12} \cdot trp \tag{8}$$

$$\frac{d5HTP}{dt} = inhib_{5HTto5HT}^{syn}\left(G_{5HT}^{*}, G_{5HT,0}^{*}\right) \cdot V_{TPH}(trp, bh4) - V_{AADC}(5HTP) \tag{9}$$

$$\begin{aligned}\frac{dc5HT}{dt} = {} & V_{AADC}(5HTP) - V_{MAT}(c5HT, v5HT) - V_{MAT}(c5HT, v5HT_r) + V_{SERT}\left(e5HT, SERT_s, K_m^{app}\right) - TC_{catab}(c5HT) \\ & - a_{15} \cdot (c5HT - e5HT)\end{aligned} \tag{10}$$

$$\begin{aligned}\frac{dv5HT}{dt} = {} & V_{MAT}(c5HT, v5HT) - a_{16} \cdot inhib_{5HTto5HT}^{R}\left(G_{5HT}^{*}, G_{5HT,0}^{*}\right) \cdot inhib_{HAto5HT}^{R}\left(G_{HA}^{\prime *}, G_{HA,0}^{\prime *}\right) \cdot fire5HT(t) \cdot v5HT \\ & + V_{traff}^{5HT}(v5HT, v5HT_0)\end{aligned} \tag{11}$$

$$\frac{dv5HT_r}{dt} = V_{MAT}(c5HT, v5HT_r) - V_{traff}^{5HT}(v5HT, v5HT_0) \tag{12}$$

$$\begin{aligned}\frac{de5HT}{dt} = {} & a_{16} \cdot inhib_{5HTto5HT}^{R}\left(G_{5HT}^{*}, G_{5HT,0}^{*}\right) \cdot inhib_{HAto5HT}^{R}\left(G_{HA}^{\prime *}, G_{HA,0}^{\prime *}\right) \cdot fire5HT(t) \cdot v5HT - V_{SERT}\left(e5HT, SERT_s, K_m^{app}\right) \\ & - a_{11} \cdot H1_{5HT}(e5HT, e5HT_0) \cdot V_{UP2}(e5HT) - a_{14} \cdot e5HT + a_8 \cdot (c5HT - e5HT) + a_9 \cdot (g5HT - e5HT)\end{aligned} \tag{13}$$

$$\frac{d5HIAA}{dt} = TC_{catab}(c5HT) + TC_{catab}(g5HT) - a_{10} \cdot 5HIAA \tag{14}$$

$$\frac{dtrp_{pool}}{dt} = V_{pool}\left(trp, trp_{pool}\right) - a_{13} \cdot trp_{pool} \tag{15}$$

$$\frac{dG_{5HT}^{*}}{dt} = a_2 \cdot (B_{5HT})^2 \cdot \left(G_{5HT}^{total} - G_{5HT}^{*}\right) - a_3 \cdot T_{5HT}^{*} \cdot G_{5HT}^{*} \tag{16}$$

$$\frac{dT_{5HT}^{*}}{dt} = a_4 \cdot (G_{5HT}^{*})^2 \cdot \left(T_{5HT}^{total} - T_{5HT}^{*}\right) - a_5 \cdot T_{5HT}^{*} \tag{17}$$

$$\frac{dB_{5HT}}{dt} = a_6 \cdot e5HT \cdot \left(B_{5HT}^{total} - B_{5HT}\right) - a_7 \cdot B_{5HT} \tag{18}$$

$$\frac{dg5HT}{dt} = a_{11} \cdot H1_{5HT}(e5HT, e5HT_0) \cdot V_{UP2}(e5HT) - TC_{catab}(g5HT) - a_9 \cdot (g5HT - e5HT) \tag{19}$$

$$\frac{dG'^{*}_{HA}}{dt} = a_{17} \cdot (B'_{HA})^2 \cdot \left(G'^{total}_{HA} - G'^{*}_{HA}\right) - a_{18} \cdot T'^{*}_{HA} \cdot G'^{*}_{HA} \tag{20}$$

$$\frac{dT'^{*}_{HA}}{dt} = a_{19} \cdot (G'^{*}_{HA})^2 \cdot \left(T'^{total}_{HA} - T'^{*}_{HA}\right) - a_{20} \cdot T'^{*}_{HA} \tag{21}$$

$$\frac{dB'_{HA}}{dt} = a_{21} \cdot eHA \cdot \left(B'^{total}_{HA} - B'_{HA}\right) - a_{22} \cdot B'_{HA} \tag{22}$$

$$\frac{dSERT_s^{pho}}{dt} = k_{ps}\left(G_{5HT}^{*}, G_{5HT,0}^{*}\right) \cdot SERT_p - k_{sp}\left(G_{5HT}^{*}, G_{5HT,0}^{*}\right) \cdot SERT_s^{pho} \tag{23}$$

$$\frac{dSERT_s}{dt} = \frac{dSERT_s^{pho}}{dt} + k_{is} \cdot SERT_i - k_{si}([ESCIT]) \cdot SERT_s \tag{24}$$

$$\frac{dSERT_p}{dt} = k_{sp}\left(G_{5HT}^{*}, G_{5HT,0}^{*}\right) \cdot SERT_p - k_{ps}\left(G_{5HT}^{*}, G_{5HT,0}^{*}\right) \cdot SERT_p \tag{25}$$

$$\frac{dSERT_i}{dt} = k_{si}([ESCIT]) \cdot SERT_s - k_{is} \cdot SERT_i \tag{26}$$

Constant parameters

Parameter	Value	Description
a_1	5	Strength of $btrp$ stabilization (h^{-1})
a_2	20	Strength of B_{5HT} to produce G_{5HT}^{*} (h^{-1})
a_3	200	Strength of T_{5HT}^{*} to deactivate G_{5HT}^{*} (h^{-1})
a_4	30	Strength of G_{5HT}^{*} to produce T_{5HT}^{*} (h^{-1})
a_5	200	Strength of T_{5HT}^{*} decay (h^{-1})
a_6	36	Strength of $e5HT$ bounding to autoreceptors (h^{-1})
a_7	20	Strength of $e5HT$ unbounding from autoreceptors (h^{-1})
a_8	1	Strength of bidirectional leakage between $c5HT$ and $e5HT$ (h^{-1})
a_9	1	Strength of bidirectional leakage between g$5HT$ and $e5HT$ (h^{-1})
a_{10}	1	Strength of catabolism of $5HIAA$ (h^{-1})
a_{11}	0.001	Uptake 2 multiplier ratio
a_{12}	2	Strength of trp removal (h^{-1})
a_{13}	1	Strength of trp_{pool} removal (h^{-1})
a_{14}	40	Strength of $e5HT$ removal (h^{-1})
a_{15}	1	Strength of replenishment of $v5HT$ from $v5HT_{reserve}$ (h^{-1})
a_{16}	1.89	Factor of release per serotonin firing event
a_{17}	100	Strength of B'_{HA} to produce G'^{*}_{HA} (h^{-1})
a_{18}	961.094	Strength of T'^{*}_{HA} to deactivate G'^{*}_{HA} (h^{-1})
a_{19}	20	Strength of G'^{*}_{HA} to produce T'^{*}_{HA} (h^{-1})
a_{20}	66.2992	Strength of T'^{*}_{HA} decay (h^{-1})
a_{21}	5	Strength of eHA bounding to heteroreceptors (h^{-1})
a_{22}	65.5179	Strength of eHA unbounding from heteroreceptors (h^{-1})
$NADPH$	330	$NADPH$ concentration (µM)
$NADP$	26	$NADP$ concentration (µM)
TRP_{in}	157.60	Rate of blood tryptophan replenishment (µM h^{-1})
G_{5HT}^{total}	10	Total G protein in serotonin autoreceptors (µM)
T_{5HT}^{total}	10	Total regulator of G protein from serotonin autoreceptors (µM)
B_{5HT}^{total}	10	Total serotonin autoreceptors (µM)

G'^{total}_{HA}	10	Total G protein in histamine heteroreceptors (µM)
T'^{total}_{HA}	10	Total regulator of G protein in histamine heteroreceptors (µM)
B'^{total}_{HA}	10	Total histamine heteroreceptors (µM)
$btrp_0$	96	$btrp$ in equilibrium (µM)
$v5HT_0$	63.05	$v5HT$ in equilibrium (µM)
$e5HT_0$	0.06	$e5HT$ in equilibrium (µM)
$G^*_{5HT,0}$	0.8561	G^*_{5HT} in equilibrium (µM)
$G'^*_{HA,0}$	0.7484	G'^*_{HA} in equilibrium (µM)
k_{is}	0.75	Strength of serotonin transporter reactivation (h^{-1})

System variables and initial conditions

Variable	Initial condition	Description
$btrp$	95.97	Blood tryptophan (µM)
$bh2$	0.0994	Cytosolic dihydrobiopterin (µM)
$bh4$	0.9006	Cytosolic tetrahydrobiopterin (µM)
trp	20.1618	Cytosolic tryptophan (µM)
$5HTP$	1.6094	Cytosolic 5-hydroxy-L-tryptophan (µM)
$c5HT$	0.0373	Cytosolic serotonin (µM)
$v5HT$	63.0383	Vesicular serotonin in readily releasable pool (RRP) (µM)
$c5HT_r$	280.0048	Reserve of vesicular serotonin (µM)
$e5HT$	0.0603	Extracellular serotonin (µM)
$5HIAA$	1.6824	5-Hydroxyindoleacetic acid (µM)
trp_{pool}	113.4099	Pool of tryptophan (µM)
G^*_{5HT}	0.8660	Serotonin activated G-protein from autoreceptors (µM)
T^*_{5HT}	1.0112	Regulator of serotonin activated G-protein from autoreceptors (µM)
B_{5HT}	0.9791	Autoreceptor bound serotonin (µM)
$g5HT$	0.0027	Glial cytosolic serotonin (µM)
G'^*_{HA}	0.7114	Histamine activated G-protein from heteroreceptors(µM)
T'^*_{HA}	1.3245	Regulator of histamine activated G-protein from heteroreceptors(µM)
B_{5HT}	1.3245	Heteroreceptor bound histamine (µM)

$SERT_s^{pho}$	0.2666	Ratio of phosphorylated serotonin transporter in the membrane surface
$SERT_s$	1.0203	Ratio of total serotonin transporter in the membrane surface
$SERT_p$	0.2297	Ratio of serotonin in vesicular pool
$SERT_i$	0	Ratio of escitalopram-induced inactive serotonin transporter

Function-defined variables

Rate of tryptophan transport from blood to cytosol (μM h^{-1})

**Function**	$$VTRP_{in}(btrp) = \frac{700 \cdot btrp}{btrp + 330}$$
**Parameters**	$V_{max} = 700\ \mu\text{M h}^{-1}, K_M = 330\ \mu\text{M}$

Rate of synthesis of 5-HTP by tryptophan hydroxylase (μM h^{-1})

**Function**	$$V_{TPH}(trp, bh4) = \frac{278 \cdot trp \cdot \frac{bh4}{20 + bh4}}{40 + trp + \frac{trp^2}{1000}}$$
**Parameters**	$V_{max} = 278\ \mu\text{M h}^{-1}, K_M^{trp} = 40\ \mu\text{M}, K_M^{bh4} = 20\ \mu\text{M}, \text{K}_\text{i} = 1000\ \mu\text{M}$

Rate of production of bh4 from bh2 by dihydropteridine reductase (μM h^{-1})

**Function**	

	$$V_{DRR}(bh2, NADPH, bh4, NADP) = \frac{5000 \cdot bh2 \cdot NADPH}{(100 + bh2) \cdot (75\ + NADPH)} - \frac{3 \cdot bh4 \cdot NADP}{(10 + bh4) \cdot (75\ + NADP)}$$
**Parameters**	$V_{max}^{forward} = 5000$ μM h^{-1}, $V_{max}^{backward} = 3$ μM h^{-1}, $K_M^{bh4} = 10$ μM, $K_M^{bh2} = 100$ μM, $K_M^{NADPH} = 75$ μM , $K_M^{NADP} = 75$ μM

Rate of trafficking between pool of tryptophan and active tryptophan in the cytosol (μM h^{-1})

**Function**	$$V_{pool}(trp, trp_{pool}) = 9 \cdot trp - 0.6 \cdot trp_{pool}$$
**Parameters**	N/A

Rate of production of c5HT from 5-HTP by aromatic amino acid decarboxylase (μM h^{-1})

**Function**	$$V_{AADC}(5HT_p) = \frac{400 \cdot 5HTP}{160 + 5HTP}$$
**Parameters**	$V_{max} = 400$ μM h^{-1}, $K_M = 160$ μM

Rate of transport of c5HT to v5HT (RRP or reserve) by vesicular monoamine transporter (μM h^{-1})

**Function**	$$V_{MAT}(c5HT, v5HT) = \frac{1230 \cdot c5HT}{0.2 + c5HT} - 1 \cdot v5HT$$ $$V_{MAT}(c5HT, v5HT_{reserve}) = \frac{1230 \cdot c5HT}{0.2 + c5HT} - 1 \cdot v5HT_{reserve}$$

**Parameters**	$V_{max} = 1230\ \mu\text{M h}^{-1}, K_M = 0.2\ \mu\text{M}$

Rate of reuptake of e5HT to c5HT by serotonin transporters (μM h^{-1})

Function

$$V_{SERT}\left(e5HT, SERT_s, K_m^{app}\right) = \frac{250 \cdot e5HT}{K_m^{app} + e5HT} \cdot SERT_s$$

$$K_M^{app}([ESCIT], K_i) = K_m\left(1 + \frac{[ESCIT]}{K_i}\right)$$

$$K_i([ESCIT]) = K_{i0} \cdot e^{-4 \cdot [ESCIT]} + K_{i,min}$$

Parameters

$V_{max} = 250\ \mu\text{M h}^{-1}, K_m = 0.06\ \mu\text{M}$
K_M^{app}: apparent K_m in the presence of escitalopram (μM)
$[ESCIT]$: concentration of escitalopram in brain (μM), calculated as $1000 \cdot\ Q_2/(V_{brain} \cdot M_r)$, where Q_2 is quantity of escitalopram in the brain (in μg), V_{brain} is volume of the mouse brain (in mL, set to 0.41 mL) and M_r is molecular weight of escitalopram (M_r = 324.39 g mol^{-1})
K_i: inhibition constant of escitalopram (μM), dependent on concentration due to allosteric biding
$K_{i,min}$: minimum K_i, set to 0.001 μM.
K_{i0}: limits value of K_i and set to 0.05 μM, so that $[ESCIT] \rightarrow 0$ then $K_i = K_{i0} + K_{i,min}$

Rate of c5HT catabolism into 5-HIAA in cytosol and glia by monoamine oxidase and aldehyde dehydrogenase (μM h^{-1})

**Function**	$TC_{catab}(c5HT) = \frac{4000 \cdot c5HT}{95 + c5HT}$ $TC_{catab}(g5HT) = \frac{4000 \cdot g5HT}{95 + g5HT}$
**Parameters**	$V_{max} = 4000\ \mu\text{M h}^{-1}, K_M = 95\ \mu\text{M}$

Rate of trafficking from vesicular reserve and readily releasable pool of vesicles (μM h^{-1})

**Function**	$V_{traff}^{5HT}(v5HT, v5HT_0) = 15 \cdot (v5HT_0 - v5HT)$
**Parameters**	N/A

Factor of uptake 2 velocity (VU2) dependent on extracellular serotonin

**Function**	$H1_{5HT}(e5HT, e5HT_0) = \begin{cases} 0, & e5HT < e5HT_0 \\ 50 \cdot (e5HT - e5HT_0), & e5HT_0 < e5HT < e5HT_0 + 0.02 \\ 1, & e5HT > e5HT_0 + 0.02 \end{cases}$
**Parameters**	N/A

Velocity of uptake 2 reuptake of e5HT into glial terminals (μM h^{-1})

**Function**	

	$$V_{UP2}(e5HT) = \frac{1700 \cdot e5HT}{0.17 + e5HT}$$
**Parameters**	$V_{max} = 1700\ \mu\mathrm{M\ h}^{-1}, K_M = 0.17\ \mu\mathrm{M}$

Rate of serotonin firing (events h^{-1})

**Function**	$$fire5HT(t) = \begin{cases} b, & t < t_{start} \\ b + r \cdot e^{-c\,(t - t_{start})}, & t_{start} < t < t_{finish} \\ b + r \cdot e^{-c\,(t - t_{start})} - e^{-c(t - t_{finish})}, & t > t_{finish} \end{cases}$$
**Parameters**	b: basal firing when no electrical stimulation is applied, set to 1 h^{-1}<br>r: strength of stimulation, set to 8 h^{-1}<br>c: dissociation constant, set to 2 h^{-1}<br>t_{start}: start of the stimulation (h)<br>t_{finish}: end of stimulation (h)<br>When a train of stimulations is applied, $t_{start} = t^0_{start} + n_{stim} \cdot t_{interval}$, where t^0_{start} is the start time of the first stimulation (h), n_{stim} is the stim number starting from 0 and $t_{interval}$ is the time between electrical stimulations (h)

Factor of inhibition of serotonin synthesis via serotonin activated G-coupled proteins

**Function**	$$inhib^{syn}_{5HTto5HT}(G^*_{5HT}, G^*_{5HT,0}) = 1 - 0.1 \cdot (G^*_{5HT} - G^*_{5HT,0})$$
**Parameters**	N/A

Factor of inhibition of serotonin release via serotonin activated G-coupled proteins

**Function**	$$inhib^{R}_{5HTto5HT}\left(G^*_{5HT}, G^*_{5HT,0}\right) = 1 - 1.5 \cdot (G^*_{5HT} - G^*_{5HT,0})$$

**Parameters**	N/A

Factor of inhibition of serotonin release via histamine activated G-coupled proteins

**Function**	$$inhib^{R}_{HAto5HT}\left(G'^{*}_{HA}, G'^{*}_{HA,0}\right) = 1 - 3 \cdot (G'^{*}_{HA} - G'^{*}_{HA,0})$$
**Parameters**	N/A

Rate of trafficking of SERTs from the vesicular pool to membrane surface (h^{-1})

**Function**	$$k_{ps}\left(G^{*}_{5HT}, G^{*}_{5HT,0}\right) = 10 + 7.5 \cdot \left(G^{*}_{5HT} - G^{*}_{5HT,0}\right)$$
**Parameters**	N/A

Rate of trafficking of SERTs from the membrane surface to the vesicular pool (h^{-1})

**Function**	$$k_{sp}\left(G^{*}_{5HT}, G^{*}_{5HT,0}\right) = 10 - 7.5 \cdot \left(G^{*}_{5HT} - G^{*}_{5HT,0}\right)$$
**Parameters**	N/A

Rate of inactivation of SERTs by escitalopram (h^{-1})

**Function**	$$k_{si}([ESCIT]) = 18.75 \cdot [ESCIT]$$
**Parameters**	$[ESCIT]$: escitalopram in the brain (μM)

Histamine terminal and glia model (Supplementary Note 4)

ODE system

$$\frac{dcHA}{dt} = inhib_{HAtoHA}^{syn}\left(G_{HA}^{*}, G_{HA,0}^{*}\right) \cdot HTDC_a \cdot V_{HTDC}(cHT) - V_{MAT}^{HA}(cHA, vHA) - V_{HNMT}(cHA) - b_1 \cdot (cHA - eHA) + V_{HAT}(eHA) - V_{MAT}^{HA}(cHA, vHA_r) \quad (27)$$

$$\frac{dvHA}{dt} = V_{MAT}^{HA}(cHA, vHA) - inhib_{HAtoHA}^{R}\left(G_{HA}^{*}, G_{HA,0}^{*}\right) \cdot activ_{5HTtoHA}^{R}\left(G_{5HT}'^{*} - G_{5HT,0}'^{*}\right) \cdot b_2 \cdot fireha(t) \cdot vHA + V_{traff}^{HA}(vHA, vHA_0) \quad (28)$$

$$\frac{dvHA_r}{dt} = V_{MAT}^{HA}(cHA, vHA_r) - V_{traff}^{HA}(vHA, vHA_0) \quad (29)$$

$$\frac{deHA}{dt} = inhib_{HAtoHA}^{R}\left(G_{HA}^{*}, G_{HA,0}^{*}\right) \cdot activ_{5HTtoHA}^{R}\left(G_{5HT}'^{*} - G_{5HT,0}'^{*}\right) \cdot b_2 \cdot fireHA(t) \cdot vHA - V_{HAT}(eHA) + b_3 \cdot (gHA - eHA) + b_1 \cdot (cHA - eHA)$$
$$-H1_{HA}(eHA) \cdot V_{HAT}^{g}(eHA) - b_4 \cdot eHA - k_{inf} \cdot V_{HAT}^{MC}(eHA) + degranulation\left(k_{inf}\right) \cdot vHA^{MC} \quad (30)$$

$$\frac{dgHA}{dt} = H1_{HA}(eHA) \cdot V_{HAT}^{g}(eHA) - b_3 \cdot (gHA - eHA) - V_{HNMT}^{g}(gHA) + \left(1 + b_{12} \cdot k_{inf}\right) \cdot HTDC_a \cdot V_{HTDC}^{g}(gHT) \quad (31)$$

$$\frac{dbHT}{dt} = HT_{in} - V_{HTL}(bHT) - V_{HTL}^{g}(bHT) - b_5(bHT - bHT_0) - k_{inf} \cdot V_{HTL}^{MC}(bHT) \quad (32)$$

$$\frac{dcHT}{dt} = V_{HTL}(bHT) - inhib_{HAtoHA}^{syn}\left(G_{HA}^{*}, G_{HA,0}^{*}\right) \cdot HTDC_a \cdot V_{HTDC}(cHT) - b_6 \cdot cHT + b_7 \cdot cHT_{pool} \quad (33)$$

$$\frac{dcHT_{pool}}{dt} = b_6 \cdot cHT - b_7 \cdot cHT_{pool} - b_8 \cdot cHT_{pool} \quad (34)$$

$$\frac{dG_{HA}^{*}}{dt} = b_{13} \cdot (B_{HA})^2 \cdot \left(G_{HA}^{total} - G_{HA}^{*}\right) - b_{14} \cdot T_{HA}^{*} \cdot G_{HA}^{*} \quad (35)$$

$$\frac{dT_{HA}^{*}}{dt} = b_{15} \cdot (G_{HA}^{*})^2 \cdot \left(T_{HA}^{total} - T_{HA}^{*}\right) - b_{16} \cdot T_{HA}^{*} \quad (36)$$

$$\frac{dB_{HA}}{dt} = b_{17} \cdot eHA \cdot \left(B_{HA}^{total} - B_{HA}\right) - b_{18} \cdot B_{HA} \tag{37}$$

$$\frac{dG'^{*}_{5HT}}{dt} = b_{19} \cdot (B'_{5HT})^2 \cdot \left(G'^{total}_{5HT} - G'^{*}_{5HT}\right) - b_{20} \cdot T'^{*}_{5HT} \cdot G'^{*}_{5HT} \tag{38}$$

$$\frac{dT'^{*}_{5HT}}{dt} = b_{21} \cdot (G'^{*}_{5HT})^2 \cdot \left(T'^{total}_{5HT} - T'^{*}_{5HT}\right) - b_{22} \cdot T'^{*}_{5HT} \tag{39}$$

$$\frac{dB'_{5HT}}{dt} = b_{23} \cdot e5HT \cdot \left(B'^{total}_{5HT} - B'_{5HT}\right) - b_{24} \cdot B'_{5HT} \tag{40}$$

$$\frac{dgHT}{dt} = V_{HTL}^{g}(bHT) - \left(1 + b_{12} \cdot k_{inf}\right) \cdot HTDC_a \cdot V_{HTDC}^{g}(gHT) - b_9 \cdot gHT + b_{10} \cdot gHT_{pool} \tag{41}$$

$$\frac{dgHT_{pool}}{dt} = b_9 \cdot gHT - b_{10} \cdot gHT_{pool} - b_{11} \cdot gHT_{pool} \tag{42}$$

$$\frac{dHTDC_a}{dt} = -\, k_{FMH}([FMH]) \cdot HTDC_a + HTDC_{in}(HTDC_a) \tag{43}$$

Constant parameters

**Parameter**	**Value**	**Description**
b_1	15	Strength of bidirectional leakage between cHA and eHA (h^{-1})
b_2	3.5	Factor of release per histamine firing event
b_3	15	Strength of bidirectional leakage between gHA and eHA (h^{-1})
b_4	0.05	Rate of eHA removal (h^{-1})
b_5	0.25	Strength of stabilization of bHT to bHT_0 (h^{-1})
b_6	2.5	Strength of trafficking from cHT to cHT_{pool} (h^{-1})
b_7	1	Strength of trafficking from cHT_{pool} to cHT (h^{-1})

b_8	1	Rate of cHT_{pool} removal for other uses (h^{-1})
b_9	1	Rate of trafficking from gHT to gHT_{pool} (h^{-1})
b_{10}	1	Rate of trafficking from gHT_{pool} to gHT (h^{-1})
b_{11}	1	Rate of gHT removal for other uses (h^{-1})
b_{12}	10	Factor of activation of gHA synthesis due to neuroinflammation
b_{13}	100	Strength of B_{HA} to produce G_{HA}^{*} (h^{-1})
b_{14}	961.094	Strength of T_{HA}^{*} to deactivate G_{HA}^{*} (h^{-1})
b_{15}	20	Strength of G_{HA}^{*} to produce T_{HA}^{*} (h^{-1})
b_{16}	66.2992	Strength of T_{HA}^{*} decay (h^{-1})
b_{17}	5	Strength of eHA bounding to autoreceptors (h^{-1})
b_{18}	65.6179	Strength of eHA unbounding from autoreceptors (h^{-1})
b_{19}	20	Strength of ${B'}_{5HT}$ to produce ${G'}_{5HT}^{*}$ (h^{-1})
b_{20}	200	Strength of $T_{5HT}^{\prime *}$ to deactivate $G_{5HT}^{\prime *}$ (h^{-1})
b_{21}	30	Strength of ${G'}_{5HT}^{*}$ to produce ${T'}_{5HT}^{*}$ (h^{-1})
b_{22}	200	Strength of $T_{5HT}^{\prime *}$ decay (h^{-1})
b_{23}	36	Strength of $e5HT$ bounding to heteroreceptors (h^{-1})
b_{24}	20	Strength of $e5HT$ unbounding to heteroreceptors (h^{-1})
G_{HA}^{total}	10	Total G protein in histamine autoreceptors (μM)
T_{HA}^{total}	10	Total regulator of G protein from histamine autoreceptors (μM)
B_{HA}^{total}	10	Total histamine autoreceptors (μM)
$G_{5HT}^{\prime total}$	10	Total G protein in serotonin heteroreceptors (μM)
${T'}_{5HT}^{total}$	10	Total regulator of G protein in serotonin heteroreceptors (μM)
$B_{5HT}^{\prime total}$	10	Total serotonin heteroreceptors (μM)
HT_{in}	636.5570	Rate of blood histidine replenishment (μM h^{-1})
bHT_0	100	bHT in equilibrium (μM)
vHA_0	63.05	vHA in equilibrium (μM)
$G_{HA,0}^{*}$	0.7484	G_{HA}^{*} in equilibrium (μM)
$G_{5HT,0}^{\prime *}$	0.8561	$G_{5HT}^{\prime *}$ in equilibrium (μM)

System variables and initial conditions

**Variable**	**Initial condition**	**Description**
cHA	3.1074	Cytosolic histamine concentration (μM)
vHA	136.3639	Vesicular histamine in readily releasable pool (μM)
vHA_r	241.9217	Reserve of vesicular histamine (μM)
eHA	1.4378	Extracellular histamine (μM)
gHA	2.0126	Glial cytosolic histamine (μM)
bHT	99.7316	Blood histidine (μM)
cHT	249.3265	Cytosolic histidine (μM)
cHT_{pool}	311.6581	Cytosolic histidine pool (μM)
G^*_{HA}	0.7114	Histamine activated G-protein from autoreceptors (μM)
T^*_{HA}	1.3245	Regulator of histamine activated G-protein from autoreceptors (μM)
B_{HA}	0.9874	Autoreceptor bound histamine (μM)
G'^*_{5HT}	0.8660	Serotonin activated G-protein from heteroreceptors (μM)
T'^*_{5HT}	1.0112	Regulator of serotonin activated G-protein from heteroreceptors (μM)
B'_{5HT}	0.9791	Heteroreceptor bound serotonin (μM)
gHT	354.6656	Glial cytosolic histidine (μM)
gHT_{pool}	177.3328	Glial cytosolic pool of histidine (μM)
$HTDC_a$	1	Ratio of active histidine decarboxylase

Function-defined variables

	Rate of transport of bHT to cHT in HA neurons (μM h^{-1})
**Function**	$$V_{HTL}(bHT) = \frac{4680 \cdot bHT}{1000 + bHT}$$
**Parameters**	$V_{max} = 4680\ \mu\text{M h}^{-1}, K_M = 1000\ \mu\text{M}$

	Rate of transport of bHT to gHT in glia (μM h^{-1})
**Function**	$$V_{HTL}^{g}(bHT) = \frac{2340 \cdot bHT}{1000 + bHT}$$
**Parameters**	$V_{max} = 4680\ \mu\text{M h}^{-1}, K_M = 1000\ \mu\text{M}$

	Rate of cHA synthesis by histidine decarboxylase in HA neurons (μM h^{-1})
**Function**	$$V_{HTDC}(cHT) = \frac{234 \cdot cHT}{270 + cHT}$$

Parameters

$V_{max} = 230\ \mu\text{M h}^{-1}, K_M = 270\ \mu\text{M}$

Rate of gHA synthesis by histidine decarboxylase in glia (μM h^{-1})

Function

$$V_{HTDC}^{g}(cHT) = \frac{61.4250 \cdot cHT}{270 + cHT}$$

Parameters

$V_{max} = 61.4250\ \mu\text{M h}^{-1}, K_M = 270\ \mu\text{M}$

Rate of transport of cHA to vHA (RRP or reserve) by monoamine transporter in HA neuron (μM h^{-1})

Function

$$V_{MAT}^{HA}(cHA, vHA) = \frac{10552 \cdot cHA}{24 + cHA} - 5 \cdot vHA$$

$$V_{MAT}^{HA}(cHA, vHA_{reserve}) = \frac{10552 \cdot cHA}{24 + cHA} - 5 \cdot vHA_{reserve}$$

Parameters

$V_{max} = 10552\ \mu\text{M h}^{-1}, K_M = 24\ \mu\text{M}$

Rate of cHA metabolism by histamine methyltransferase in HA neuron (μM h^{-1})

Function

	$$V_{HNMT}(cHA) = \frac{185.5 \cdot cHA}{4.2 + cHA}$$
**Parameters**	$V_{max} = 185.5\ \mu\text{M h}^{-1}, K_M = 4.2\ \mu\text{M}$

Rate of gHA metabolism by histamine methyltransferase in glia (μM h^{-1})

**Function**	$$V_{HNMT}^{g}(cHA) = \frac{212 \cdot cHA}{4.2 + cHA}$$
**Parameters**	$V_{max} = 185.5\ \mu\text{M h}^{-1}, K_M = 4.2\ \mu\text{M}$

Rate of eHA reuptake by the putative HA transporter into HA neuron (μM h^{-1})

**Function**	$$V_{HAT}(eHA) = \frac{4128.3 \cdot eHA}{10 + eHA}$$
**Parameters**	$V_{max} = 4128.3\ \mu\text{M h}^{-1}, K_M = 10\ \mu\text{M}$

Rate of eHA reuptake by the putative HA transporter into glia (μM h^{-1})

**Function**	$$V_{HAT}^{g}(eHA) = \frac{13500 \cdot eHA}{10 + eHA}$$
**Parameters**	

$V_{max} = 13500\ \mu\text{M h}^{-1}, K_M = 10\ \mu\text{M}$

Rate of trafficking from histamine vesicular reserve and readily releasable pool of vesicles (μM h^{-1})

**Function**	$$V_{traff}^{HA}(vHA, vHA_0) = \ 15 \cdot (vHA - vHA_0)$$
**Parameters**	N/A

Factor of glia histamine reuptake dependent on eHA

**Function**	$$H1_{HA}(eHA) = \begin{cases} 0.025, & eHA < 0.025 \\ 0.1 \cdot (e5HT - 19), & 0.025 < eHA < 29 \\ 1, & e5HT > 29 \end{cases}$$
**Parameters**	N/A

Rate of histamine firing (events h^{-1})

**Function**	$$fireHA(t) = \begin{cases} b, & t < t_{start} \\ b + r \cdot e^{-c\,(t - t_{start})}, & t_{start} < t < t_{finish} \\ b + r \cdot e^{-c\,(t - t_{start})} - e^{-c(t - t_{finish})}, & t > t_{finish} \end{cases}$$
**Parameters**	b: basal firing when no electrical stimulation is applied, set to 1 event h^{-1}<br>r: strength of stimulation, set to 150 event h^{-1}<br>c: dissociation constant, set to 2 h^{-1}<br>t_{start}: start of the stimulation (h)

t_{finish}: end of stimulation (h)<br>When a train of stimulations is applied, $t_{start} = t_{start}^{0} + n_{stim} \cdot t_{interval}$, where t_{start}^{0} is the start time of the first stimulation (h), n_{stim} is the stim number starting from 0 and $t_{interval}$ is the time between electrical stimulations (h)

Factor of inhibition of histamine synthesis via histamine activated G-coupled proteins

**Function**	$$inhib_{HAtoHA}^{syn}\left(G_{HA}^{*}, G_{HA,0}^{*}\right) = 1 - 0.1 \cdot (G_{HA}^{*} - G_{HA,0}^{*})$$
**Parameters**	N/A

Factor of inhibition of histamine release via histamine activated G-coupled proteins

**Function**	$$inhib_{HAtoHA}^{R}\left(G_{HA}^{*}, G_{HA,0}^{*}\right) = 1 - 2 \cdot (G_{HA}^{*} - G_{HA,0}^{*})$$
**Parameters**	N/A

Factor of activation of histamine release via serotonin activated G-coupled proteins in heteroreceptors

**Function**	$$activ_{5HTtoHA}^{R}\left(G_{5HT}'^{*} - G_{5HT,0}'^{*}\right) = 1 + 3 \cdot (G'^{*}_{5HT} - G'^{*}_{5HT,0})$$
**Parameters**	N/A

Inactivation speed of histidine decarboxylase by FMH inhibition (h^{-1})

**Function**	$$k_{FMH} = k_1 \cdot \frac{k_2}{k_2 + k_3} \cdot \frac{[FMH]}{K_i + [FMH]}$$

**Parameters**	$k_1 \cdot \frac{k_2}{k_2+k_3}$: inactivation rate (set to 10.40 h^{-1}). k_1 is the rate of FMH biding to the enzyme (h^{-1}), k_2 is the rate of FMH covalently biding to histidine decarboxylase (h^{-1}) and k_3 is the rate of FMH becoming non-reactive and not binding covalently to the enzyme (h^{-1})<br>$[FMH]$: concentration of FMH in µM<br>K_i: dissociation constant of the FMH-HTDC complex (set to 8.3 µM)

	**Replenishment of histidine decarboxylase by protein synthesis (h^{-1})**
**Function**	$$HTDC_{in}(HTDC_a) = 0.55 \cdot (1 - HTDC_a)$$
**Parameters**	$HTDC_a$: ratio of active histidine decarboxylase.

Mast cell model (Supplementary Note 5)

ODE system

$$\frac{dcHT^{MC}}{dt} = k_{inf} \cdot V_{HTL}^{MC}(bHT) - HTDC_a \cdot V_{HTDC}^{MC}(cHT^{MC}) - c_1 \cdot cHT^{MC} + c_2 \cdot cHT_{pool}^{MC} \quad (44)$$

$$\frac{dcHT_{pool}^{MC}}{dt} = c_1 \cdot cHT^{MC} - c_2 \cdot cHT_{pool}^{MC} - c_3 \cdot cHT_{pool}^{MC} \quad (45)$$

$$\frac{dcHA^{MC}}{dt} = HTDC_a \cdot V_{HTDC}^{MC}(cHT^{MC}) - V_{MAT,HA}^{MC}(cHA^{MC}, vHA^{MC}) - V_{HNMT}^{MC}(cHA^{MC}) + k_{inf} \cdot V_{HAT}^{MC}(eHA) \tag{46}$$

$$\frac{dvHA^{MC}}{dt} = V_{MAT,HA}^{MC}(cHA^{MC}, vHA^{MC}) - degranulation\left(k_{inf}\right) \cdot vHA^{MC} \tag{47}$$

Constant parameters

Parameter	Value	Description
c_1	1	Strength of trafficking from cHT^{MC} to cHT_{pool}^{MC} (h^{-1})
c_2	1	Strength of trafficking from cHT_{pool}^{MC} cHT^{MC} to (h^{-1})
c_3	1	Rate of cHT^{MC} removal for other uses (h^{-1})

System variables and initial conditions

Variable	Initial condition	Description
cHT^{MC}	350	Cytosolic histidine in mast cells (µM)
cHT_{pool}^{MC}	150	Cytosolic histidine pool in mast cells (µM)
cHA^{MC}	3	Cytosolic histamine in mast cells (µM)
vHA^{MC}	140	Vesicular histamine in mast cells (µM)

Function-defined variables

	Rate of transport of bHT to cHT in mast cells (µM h^{-1})
**Function**	

	$$V_{HTL}^{MC}(bHT) = \frac{109.5 \cdot bHT}{1000 + bHT}$$
**Parameters**	$V_{max} = 219\ \mu\text{M h}^{-1}, K_M = 1000\ \mu\text{M}$

Rate of gHA synthesis by histidine decarboxylase in glia (μM h^{-1})

**Function**	$$V_{HTDC}^{MC}(cHT^{MC}) = \frac{877.50 \cdot cHT^{MC}}{270 + cHT^{MC}}$$
**Parameters**	$V_{max} = 877.50\ \mu\text{M h}^{-1}, K_M = 270\ \mu\text{M}$

Rate of transport of cHA to vHA by monoamine transporter in mast cell (μM h^{-1})

**Function**	$$V_{MAT,HA}^{MC}(cHA^{MC}, vHA^{MC}) = \frac{21104 \cdot cHA^{MC}}{24 + cHA^{MC}} - 5 \cdot\ vHA^{MC}$$
**Parameters**	$V_{max} = 21104\ \mu\text{M h}^{-1}, K_M = 24\ \mu\text{M}$

Rate of cHA metabolism by histamine methyltransferase in mast cells (μM h^{-1})

**Function**	$$V_{HNMT}^{MC}(cHA^{MC}) = \frac{21.20 \cdot cHA^{MC}}{4.2 + cHA^{MC}}$$

Parameters

$V_{max} = 21.20\ \mu\text{M h}^{-1}, K_M = 4.2\ \mu\text{M}$

Rate of eHA reuptake by the putative HA transporter into glia (μM h^-1)

Function

$$V_{HAT}^{MC}(eHA) = \frac{3375 \cdot eHA}{10 + eHA}$$

Parameters

$V_{max} = 3375\ \mu\text{M h}^{-1}, K_M = 10\ \mu\text{M}$

Factor of activation of neuroinflammation: synthesis and release of histamine by glia and mast cells (μM h^-1)

Function

$$k_{inf}(t, switch_{inf}, t_{start}) = \begin{cases} 0.001 \cdot switch_{inf} & t < t_{start} \\ \dfrac{switch_{MC}}{1 + e^{-20(t - t_h)}} & t > t_{start} \end{cases}$$

$$t_h = t_{start} + \frac{\ln(999)}{20}$$

Parameters

t_{start}: start time of neuroinflammation processes (h)
t_h: time at which the sigmoid function is at 50% its capacity, so that k_{inf} = 0 (h)
$switch_{inf}$: boolean (takes the value of 0 or 1) which activates or deactivates the process of histamine turnover from mast cells and glia

	Factor of degranulation strength dependent on activation of degranulation (μM h^{-1})
**Function**	$$degranulation\left(k_{inf}\right) = 3 \cdot k_{inf}$$
**Parameters**	k_{inf}: factor of activation of synthesis and reléase of histamine (see function above)

Supplementary figures

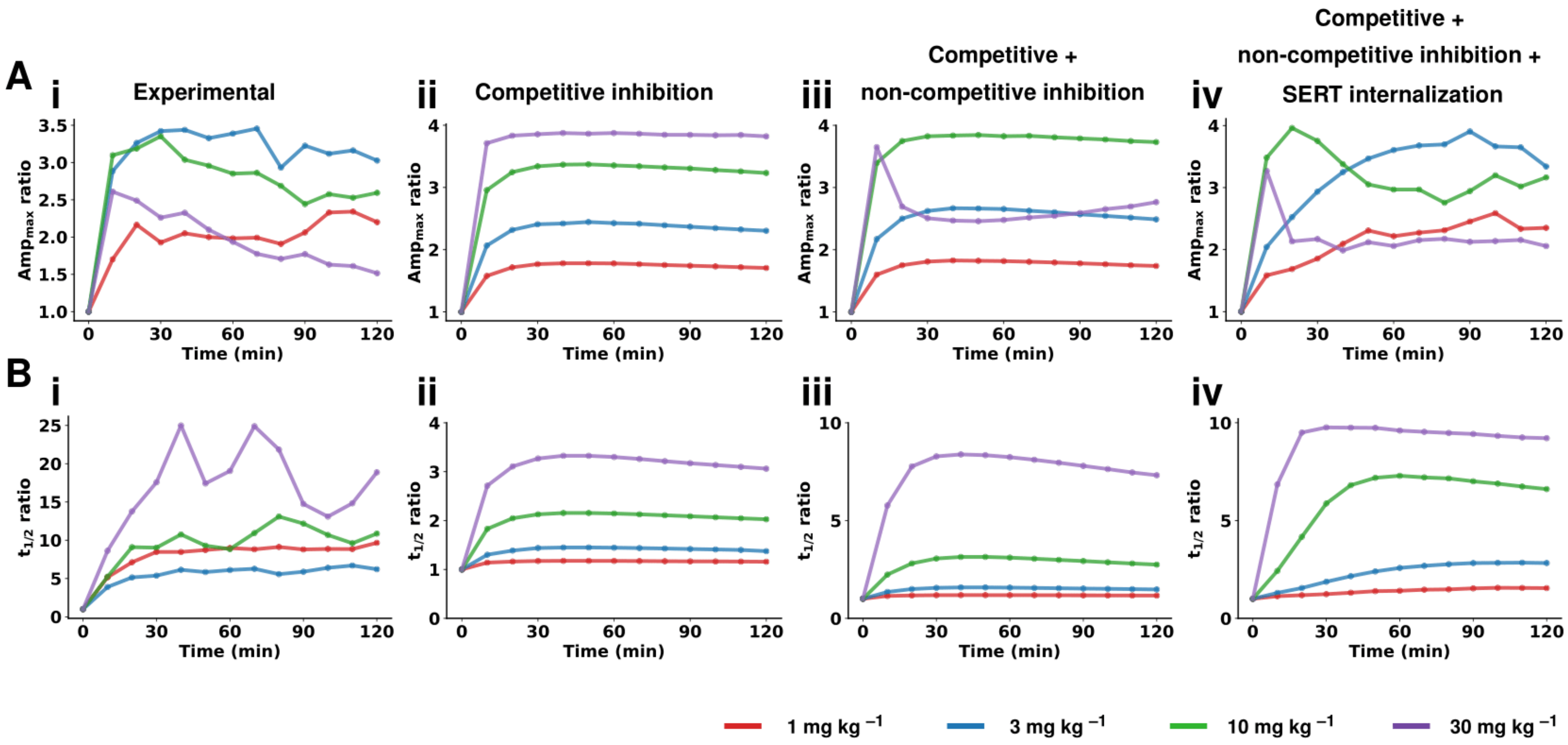

Figure S1. Maximum Amplitude and Clearance of Experimental and Simulated FSCV Traces. (A) Ratio of maximum amplitude respect to control (pre-drug administration) over time for the (i) experimental traces, (ii) traces simulated including escitalopram competitive inhibition, (iii) Simulated traces adding escitalopram non-competitive inhibition and (iv) simulated traces adding SERT internalization. (B) Ratio of half-life of evoked serotonin respect to control (pre-drug administration) over time for the same conditions given in panel A. Doses given and simulated are provided in the legend.

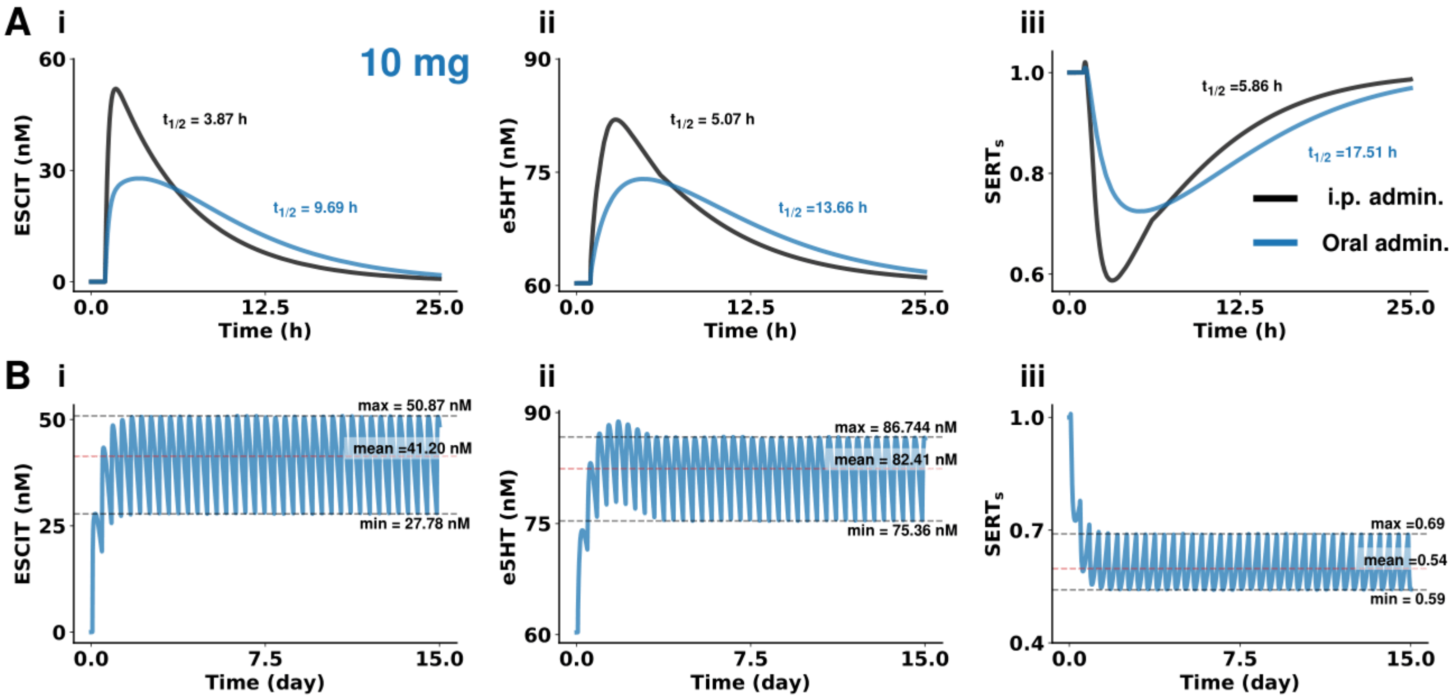

Figure S2: Chronic Oral Administration of 10 mg Escitalopram. (A) Modelling simulations illustrating brain concentrations of escitalopram (i), serotonin (ii), and SERT surface ratio (iii) following intraperitoneal (i.p.) injection or oral administration of 2.06 mg kg^{-1} (equivalent to a 10 mg human dose). The provided data includes clearance half-lives. (B) Simulation depicting the impact of chronic oral dosing on escitalopram (i), serotonin (ii), and SERT surface ratio (iii). Administration is repeated every 8 hours, mirroring the human daily dose regimen. Panels present the oscillation's maximum and minimum values, as well as the mean cumulative concentration.

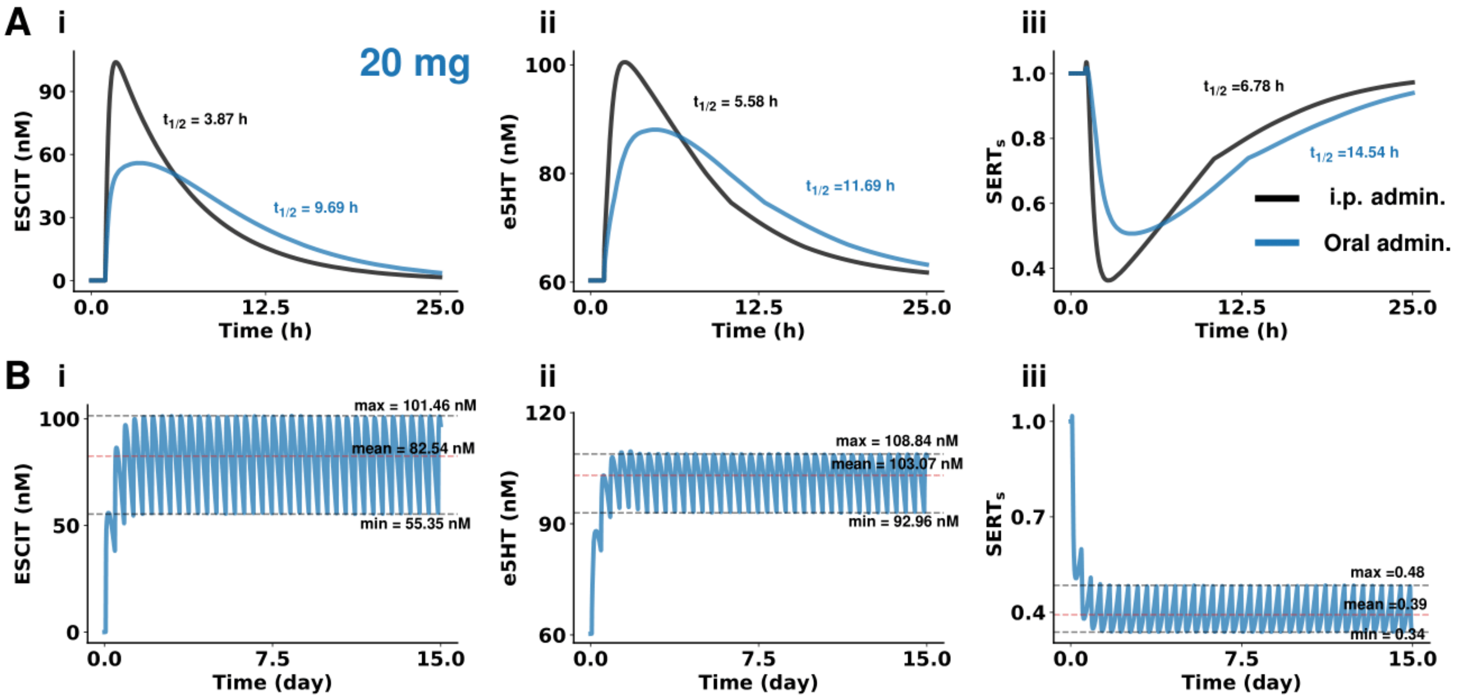

Figure S3: Chronic Oral Administration of 20 mg Escitalopram. (A) Modelling simulations illustrating brain concentrations of escitalopram (i), serotonin (ii), and SERT surface ratio (iii) following intraperitoneal (i.p.) injection or oral administration of 4.11 mg kg^{-1} (equivalent to a 20 mg human dose). The provided data includes clearance half-lives. (B) Simulation depicting the impact of chronic oral dosing on escitalopram (i), serotonin (ii), and SERT surface ratio (iii). Administration is repeated every 8 hours, mirroring the human daily dose regimen. Panels present the oscillation's maximum and minimum values, as well as the mean cumulative concentration.

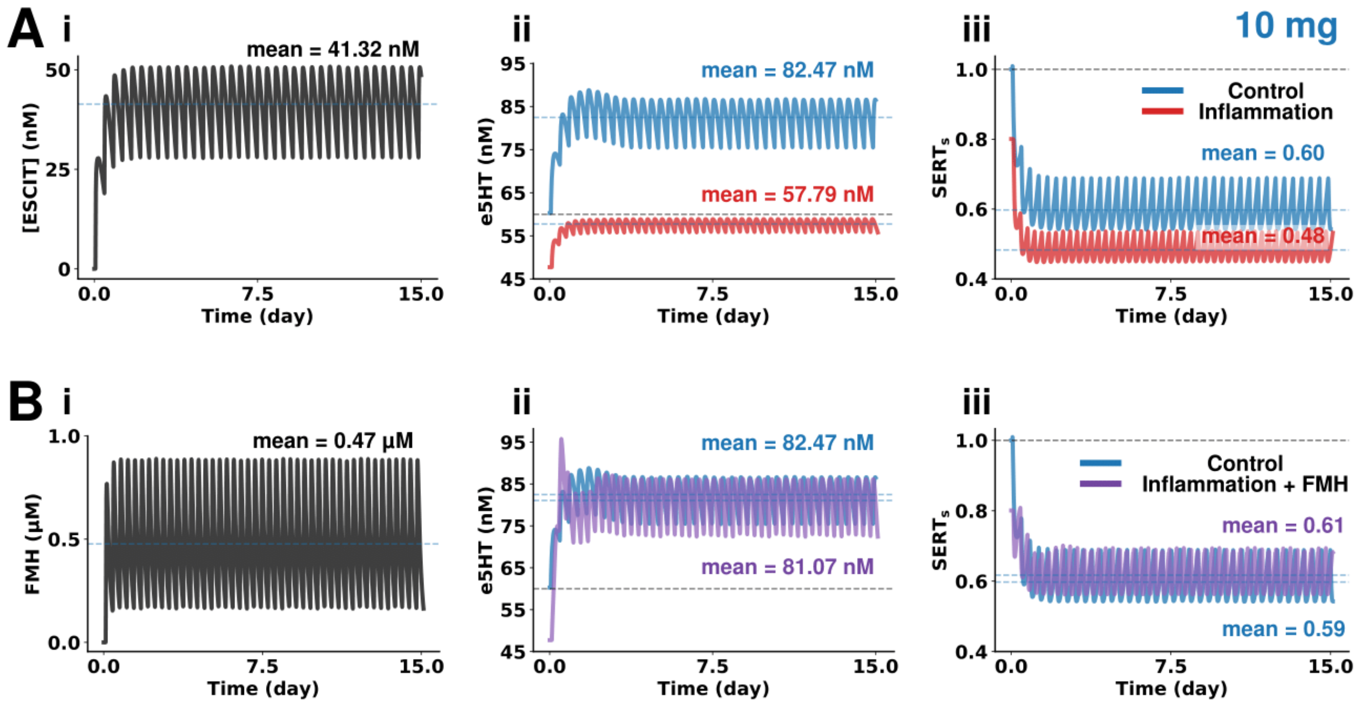

Figure S4: Impact of Oral Chronic Administration of 10 mg Escitalopram During Inflammation. (A) Modelling results depicting the brain concentrations of escitalopram (i), serotonin (ii), and the SERT surface ratio (iii) during oral chronic dosing of escitalopram (equivalent to a 10 mg pill or ~2.06 mg kg^{-1}) in both the control state and in the presence of inflammation. The inflammation simulation involves the release of histamine triggered by mast cells and glia 35 days prior to the first dose, followed by administration every 8 hours. (B) Modelling outcomes of oral chronic co-administration of FMH (equivalent to a 2.5 mg human dose for mice or ~0.51 mg kg^{-1}) and escitalopram (at the same dose as in A) during inflammation (shown in purple), compared to control escitalopram administration as outlined in panel A (shown in blue).

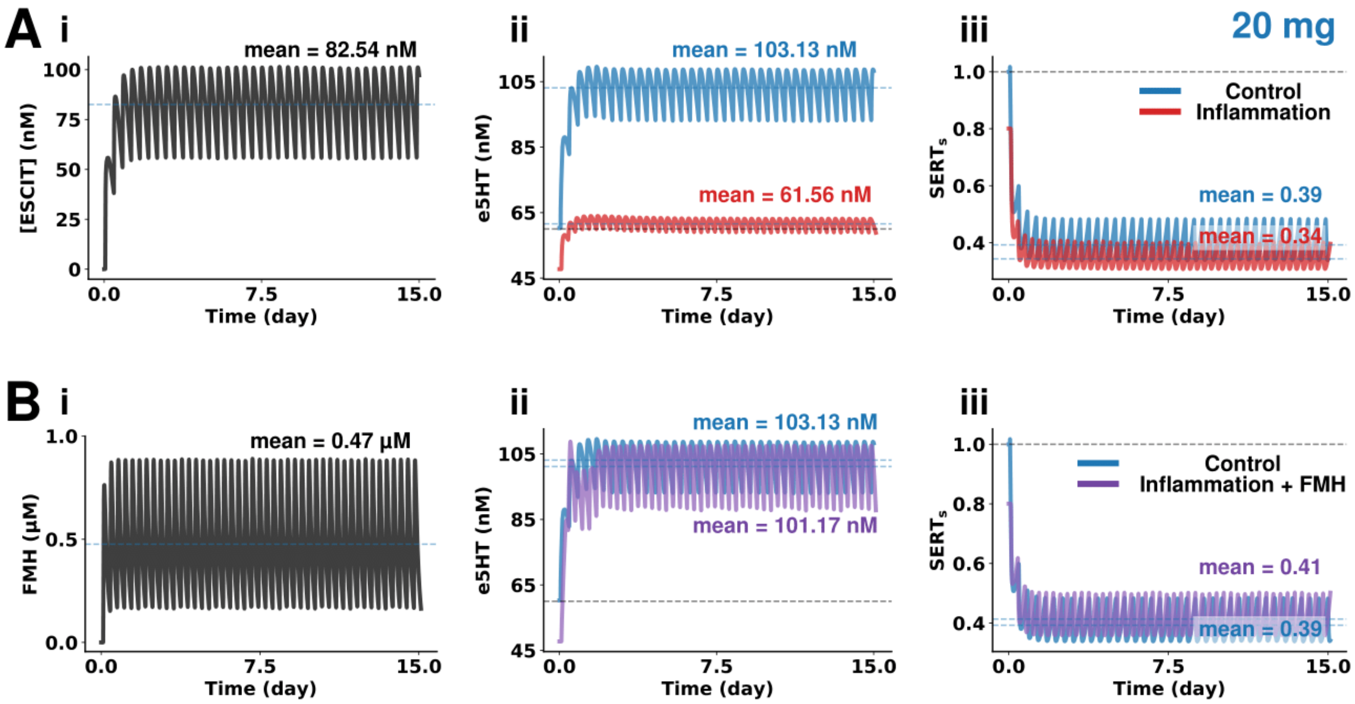

Figure S5: Impact of Oral Chronic Administration of 20 mg Escitalopram During Inflammation. (A) Modelling results depicting the brain concentrations of escitalopram (i), serotonin (ii), and the SERT surface ratio (iii) during oral chronic dosing of escitalopram (equivalent to a 20 mg pill or ~4.11 mg kg^{-1}) in both the control state and in the presence of inflammation. The inflammation simulation involves the release of histamine triggered by mast cells and glia 35 days prior to the first dose, followed by administration every 8 hours. (B) Modelling outcomes of oral chronic co-administration of FMH (equivalent to a 2.5 mg human dose for mice or ~0.51 mg kg^{-1}) and escitalopram (at the same dose as in A) during inflammation (shown in purple), compared to control escitalopram administration as outlined in panel A (shown in blue).
